# Supplementary material for: Fast and Efficient Simulation of the FEBID Process with Thermal Effects
Source: Nanomaterials (Basel). 2023 Feb 25;13(5):858. doi: 10.3390/nano13050858 (PMC10005571; doi:10.3390/nano13050858)
Supplement: Supplementary file 1 [file nanomaterials-13-00858-s001.zip › nanomaterials-2227929-supplementary.pdf]

nanomaterials

Supplementary Materials

Fast and Efficient Simulation of the FEBID Process with Thermal Effects

Alexander Kuprava and Michael Huth *

Institute of Physics, Goethe University, 60438 Frankfurt am Main, Germany
* Correspondence: michael.huth@physik.uni-frankfurt.de

S1. 3D space and data

Based on Cartesian coordinates, the discretized space is divided into blocks/voxels or cells with an edge length c that composes a 3D domain of cells extending in x, y and z directions. The cell size defines the discretization of the simulation volume and the resolution of the final object and influences simulation speed and time step restrictions for the numerical solution of reaction–diffusion equations and heat transfer equations. The 3D data is stored in arrays of the same size and shape with various datasets (*solid structure, surface deposit, precursor coverage, SE surface flux, temperature, surface temperature, residence time, diffusion coefficient*). Each dataset can be rendered by hiding empty cells and optionally coloring non-empty cells. Eventually, every dataset represents a layer that can be superimposed or swapped for a demonstrative comparison as shown in Figure S1.

All datasets have a designated relation to each other that arises from the cell types. A single cell can correspond to several datasets at the same time and hold several different values. Thus, datasets may have common cells and overlap. The cell properties are described in the next section, while here, we focus on the data framework. Datasets are defined by the data they represent and a shape or a profile that is defined by the positions or 3D arrangement of the cells it contains. The datasets mentioned above have the following relation: the solid structure and the temperature profile have the same shape; the surface deposit has the same shape as the surface profile. Precursor coverage, residence time profile, diffusion coefficient profile and surface temperature shape are a sum of surface and semi-surface profiles.

The collection of datasets or full structure snapshots is saved regularly with embedded current simulation time and beam position. Additionally, initially used parameter sets (precursor properties, gas flux and beam settings) are saved to a file as well. Thus, the deposition process state can be retrieved and inspected at any stage.

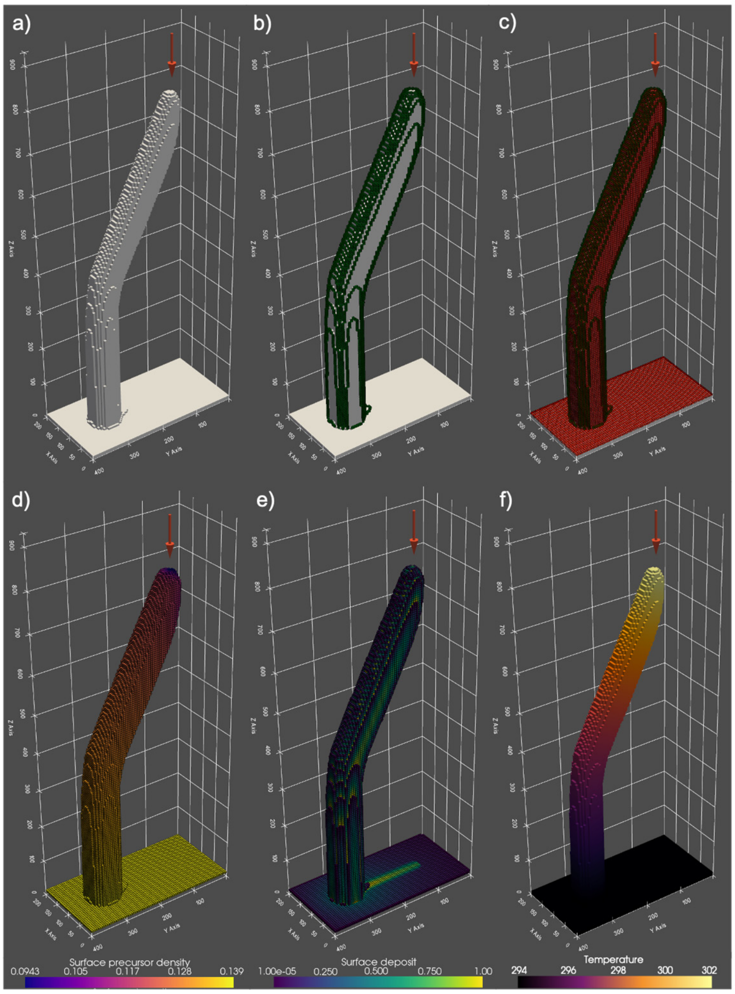

Figure S1. Representations of the 3D structure data: a) solid fully-filled cells; b) semi-surface cells superimposed onto the solid structure; c) semi-surface and surface cells superimposed onto the solid structure; d) precursor density distribution on the surface; e) partially filled surface cells representing surface deposit; and f) temperature profile. The arrow depicts the beam position.

S2. Cellular Evolution

At each timestep Δt, the reaction–diffusion equation is solved, and a miniscule amount of deposit is added to the irradiated surface cells. The values of the deposited volume contained in the cells represent a normalized fractional value with unity at maximum. When a cell is filled up to the unity, a surface update event occurs. The algorithm associated with the event updates the local configuration of the cell types.

There are five types of cells that are used to implement the deposition process and surface evolution. The first type is *void*—an empty voxel that corresponds to the gas phase. The second type is a *surface cell*. These are the cells that contain a precursor density and a deposited volume value. Accordingly, the reaction–diffusion equation with the four terms (adsorption, desorption, diffusion and dissociation) is solved for the whole domain of surface cells. Surface cells are the only type of cells that can be filled with deposits if exposed to SE flux. The next type is called *semi-surface cell.* They represent one of the auxiliary types. Despite having ‘surface’ in their name, these cells do not contain deposits and cannot be involved in adsorption/desorption processes. Their purpose is to serve as a connection between the surface cells that are disconnected from each other by a step. Thus, they are only considered during the solution of reaction–diffusion equation (Section 3). The last type of cells is the *ghost cell*. Ghost cells are a supporting type of cell that is used only during the solution of reaction–diffusion equation that makes sure that diffusion only occurs along the surface, i.e., via surface and semi-surface cells.

There are several rules that define the grid structure and dictate the evolution of the surface. They arise from the following possible surface configurations:

1. If a cell has a neighboring solid cell, it must be a surface cell;
2. If a cell is void and neighbors two or three surface cells, it must be a semi-surface cell;
3. If a cell is solid or void and neighbors at least one surface cell, it must be a ghost cell;
4. When a surface cell is fully filled with a deposit, it becomes a solid cell.

In case of a surface evolution event, i.e., if a surface cell is filled to above unity, its surplus deposit is distributed evenly among the newly defined surface cells. The precursor density in these new cells is copied from the cell that was previously filled, while if a semi-surface cell is converted into a surface cell, the current precursor density in that cell is preserved.

The rules describe a rather simple and robust model of surface evolution. In particular, it does not require the time-consuming calculation of a normal to the surface. These rules not only define the cell evolution mechanism but also allow us to determine all the cells from a single data domain of solid cells. When a cell is filled and the surface update is queued, the processing algorithm requires a domain containing the 2nd nearest neighbors around at the filled cell, which results in a compact 5 × 5 × 5 array (Figure S2). This algorithm allows for a quick cell evolution processing that does not depend on the total grid size, i.e., the processing speed remains the same for a grid of any size.

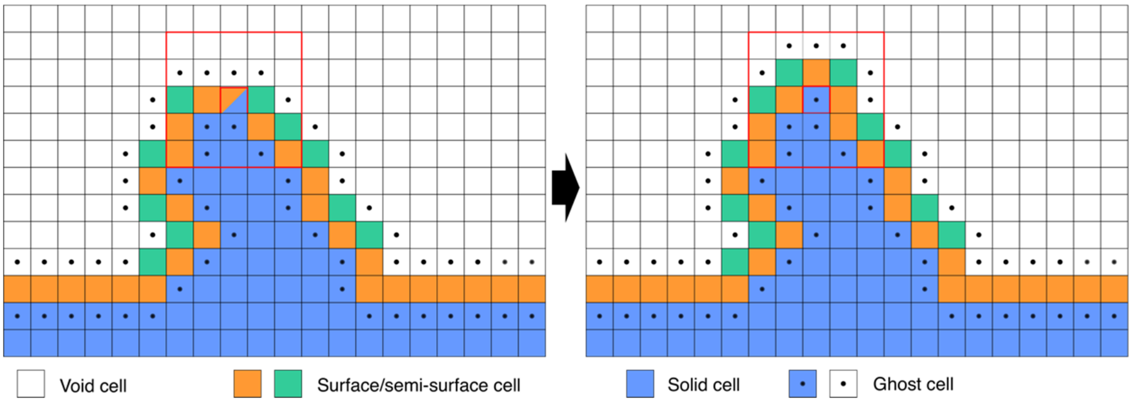

Figure S2. Schematic representation of a valid cell structure and cell evolution. The filled cell and its second nearest neighbors are marked with a red frame. The diagonally divided cell on the left scheme is half-filled. The cell at the same position on the right scheme is filled and its neighboring cell types reassigned according to the described cell evolution rules.

S3. Diffusion

In the FEBID process, diffusion is one of the four processes that influence surface precursor coverage and, consequently, the deposition process. The migration of the precursor molecules takes place exclusively at the surface, which forbids diffusive flow into the gas phase and the solid or normal to the surface. In our simulation, we use a rather simple finite difference FTCS scheme coupled with the Runge–Kutta 4 method for improved accuracy. The restriction of diffusion to the surface is realized via the ghost-cell method, as detailed above.

The FCTS scheme can be represented as a stencil operator that is applied to every surface cell, as schematically shown in Figure S3. The problem that arises from a single-cell thick surface is that steps on the voxelized surface break the continuity of the surface. During stencil operations, these cells effectively remain out of the reach of the operator. As introduced in Section S2, semi-surface cell species are introduced to the data framework to support diffusion along the whole surface. Another feature introduced was the application of the Runge–Kutta method. Besides increased numerical accuracy, it increased the effective radius of the Laplacian operator from one to four cells.

In order to enable diffusion exclusively along the surface, the so-called 'ghost cell' method is used. The thin layer of surface cells is ultimately contained between the void and solid cells. The solid and void cells that neighbor a surface cell are marked as ghost cells, encapsulating the whole surface (Figure S2). During the solution of the reaction–diffusion equation, the precursor density within a ghost cell $n_{i+1,j,k}$ 'mirrors' the value of the neighboring surface cell $n_{i,j,k}$, effectively setting the diffusive flux J between them to zero:

$$n_{i,j,k} = n_{i+1,j,k} \quad \Rightarrow \quad J = 0$$

Applying this operation to all the ghost cells guarantees diffusive flux only along the surface by applying Dirichlet conditions at the surface boundary.

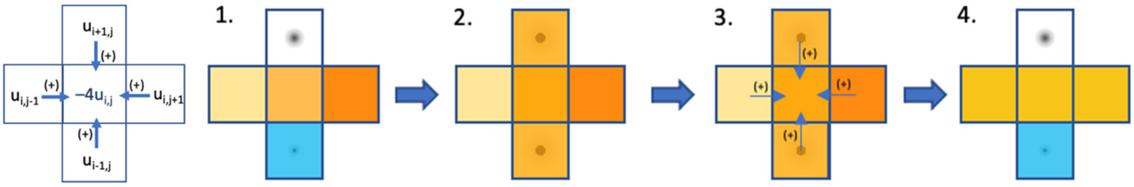

Figure S3. Diffusion calculation scheme for 2D case: leftmost is the base stencil operator. Application of the stencil with the ghost-cell method: 1) initial state, 2) values of the current cell are copied to the neighboring ghost cells, 3) stencil operator is applied, and 4) restored values of the ghost cells.

S4. Heat Transfer

As an electron beam traverses the surface, it inevitably causes the heating of the sample due to various inelastic processes. As a result, the temperature of the sample rises depending on its size, geometry and thermal properties of the material the sample is made of. The heat transfer process is described by a parabolic partial differential equation and can be numerically solved by the same methods as the reaction–diffusion equation. Note that due to a significantly smaller characteristic time scale, heat transfer occurs at time steps orders of magnitude shorter than the ones suitable for describing diffusion. A Simultaneous Over-Relaxation (SOR) method is used to calculate the temperature profile of the sample in stationary state, which arises from the steady-state nature of heat transfer in our model [20].

The temperature profile is calculated when at least two layers of cells are fully filled and then readjusted at a certain frequency. The time interval length is dependent on the total deposited volume increase as a generic growth progression characteristic. In between the intervals, the filled cells are assigned the average temperature of their closest neighbors. Due to the relaxation nature of the applied solution, the greater the temperature difference between consecutive readjustments, the longer the computation time. We have observed that using smaller intervals does not lead to a proportional computation time increase but only to an increase of several percent. Thus, increasing the frequency of determining the temperature profile to a certain point does not lead to sizable performance issues but improves time resolution. We identified an optimum interval value associated with a deposit volume increase of 10'000 nm^3, which effectively results in 0.05 K resolution for the thinnest structures (50 nm).

The calculated temperature profile refers to the solid cells. For the determination of the diffusion coefficient and residence time, the surface cell temperature distribution is required. It is estimated by averaging over the temperatures in the respective neighboring solid cells. The last step is calculation of the surface diffusion coefficient and residence time for each individual surface cell. As these properties are no longer constant and have to correspond to the temperature in each cell, they are calculated as 3D profiles as well matching on the surface profile.

The solution of the steady-state temperature profile is done via the Simultaneous Over-Relaxation method [41]. In general, a given PDE is solved via the FTCS scheme with the maximum time step without the time differential. The solution is considered converged when the norm between the current and previous step is less or equal to the target accuracy. In practice, accuracy evaluation is several times more computationally expensive than a solution step.

In our simulation, a prediction algorithm is applied to avoid unnecessary accuracy evaluations. The propagation of the achieved accuracy has been observed to exhibit an exponential convergence behavior regardless of the structure shape. By calculating the norm ε between two consecutive solutions three times and fitting data to an exponential equation versus the number of iterations n, it is possible to predict the number of iterations required to achieve the target accuracy:

$$\varepsilon(n) = a \cdot \exp(b \cdot n)$$

If the solution does not converge at first prediction, the norm is calculated again, and the exponential curve is refitted. By using such progressive fitting, the solution can be reliably found with just several accuracy evaluations. The number of excessive iterations may reach 6% but decreases with an increased number of required iterations.

S5. Cell Traversal

The discretization of the simulation volume prompts a method to determine the coordinates where the voxels are entered and exited by the electrons. As the electron travels through the discretized space, it is essential to know the coordinates in order to determine the length intersected and the type of the voxel crossed. This problem is known as ray tracing. We are using the Accelerated Ray-Tracing System [42,43] for the iterative determination of intersection coordinates.

An example of a ray on a grid is shown in Figure S4. The propagation through the grid and the intersection of the voxels is characterized by the consecutive crossing of evenly-spaced planes (or lines in 2D) that form the grid. Across the ray's path, the number of intersected voxels is equal to the sum of the vertical and horizontal lines crossed. These crossing points are regularly and evenly spaced along the corresponding axis and their positions depend on the ray direction and grid spacing. Variables t_x and t_y are the fractions of the total distance that the ray has reached at a certain point along each axis. Further on, t_x and t_y are referred to as a vector t, and all the variables are considered 2D vectors for clarity, although the solution is applied in 3D within the growth simulation. The fractional distance t is calculated by dividing the traversed distance d by the total distance l; thus, the further the ray has propagated, the closer the t is to unity:

$$t = \frac{d}{l}$$

The vector t can be calculated once and then incremented as the ray travels through the voxels. The initial t_0 is equal to the fraction of the remaining distance in the initial cell:

$$t_0 = mod\left(\frac{p_0}{c}\right)$$

In order to iterate through the consecutive values of t that correspond to the crossing points, an increment Δt is to be defined. It can be calculated by dividing the grid spacing c by the total distance l:

$$\Delta t = \frac{c}{l}$$

It should be noted that these operations are done component-wise. The more grid lines the ray crosses along its path, the smaller the increment Δt.

$$t_{n+1} = t_n + \Delta t = \frac{d + \Delta d}{l}$$

The t component is incremented by a corresponding increment component only if it is currently the smallest and only one component at a time. When all components of the t are equal or more than unity, the ray has traveled its full path.

In this manner, by calculating the initial t and iterating using the increment Δt, the grid can be traversed in a very simple and effective manner. The position of every single intersection point can be calculated as:

$$p = p_0 + \min(t) \cdot l,$$

where p is the current crossing point, *min(t)* corresponds to the smallest component of t. If t exceeds unity, the end-point of the ray p_n is taken.

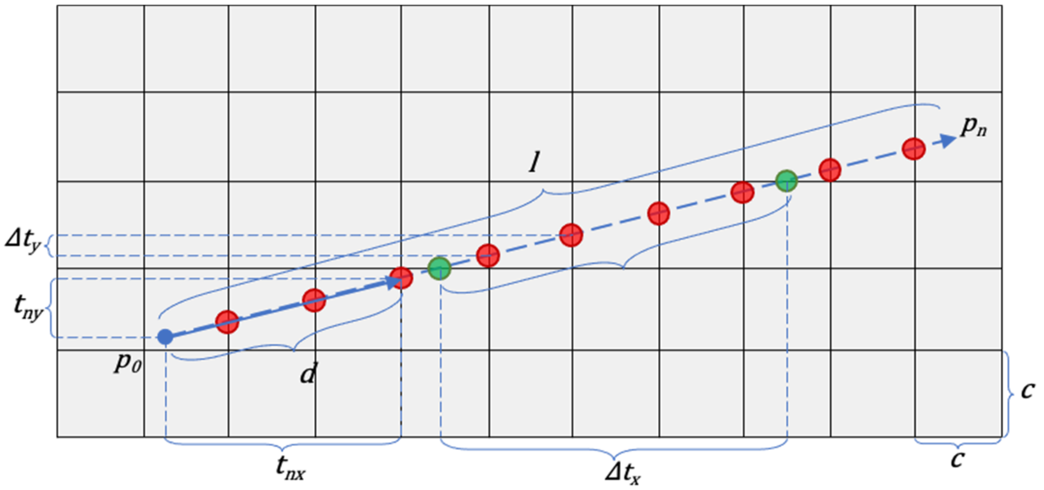

Figure S4. Intersection with the cells along the x-axis are marked red and along the y-axis they are marked green.

The general algorithm can be represented as shown in the snippet, where p_0, c and l are given quantities:

```
step_t = g/l
t = mod(p0, c)
  while True:
    index = min(t) # a function that returns the position of the
smallest value
    if t[index] > 1:
      break
    p = p0 + t[index] · l
    t[index] += step_t[index]
```

The crossing coordinates are saved and used to calculate deposited energy in Monte Carlo simulations. Additionally, the algorithm can be modified to detect a collision with a specific cell type to identify intersections of solid or surface cells.

S6. Parameter Influence

During the process of systematically studying the influence of precursor parameters' values, several iterative approximations have to be made concerning the value of activation energies used to calculate residence time and surface diffusion coefficient. The data collected from the executed simulations shed light on a number of structural features which are affected most by these parameters. As seen from Figure S5, parameters have different impacts on the resulting deposit features, including height, segment angle, thickness, total volume and local co-deposit.

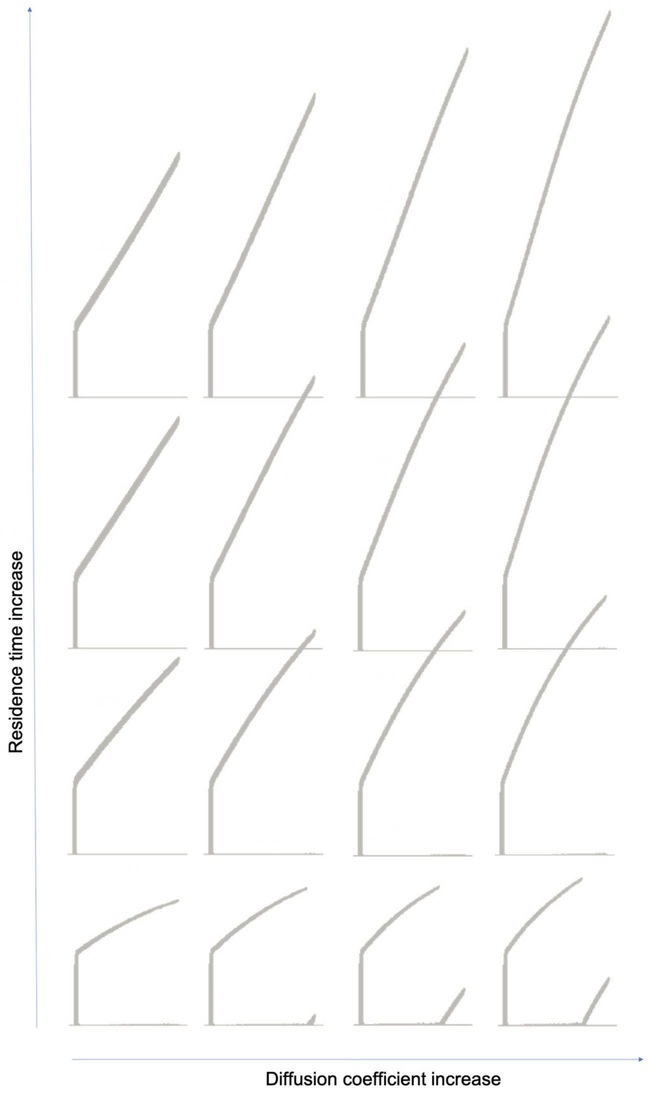

Figure S5. Influence of the residence time and the surface diffusion coefficient on the hockey stick shape. Variation of these parameters was achieved by changing the respective activation energies (eq. 3 and 4 in the main text).
